# Patients diabétiques de type 2 hypertendus: préfèrent-ils traiter le diabète ou l'hypertension artérielle?

**DOI:** 10.11604/pamj.2014.17.193.3439

**Published:** 2014-03-13

**Authors:** Hind El Aassri, Ghizlane El Mghari, Nawal El Ansari

**Affiliations:** 1 Service d'Endocrinologie, Diabétologie et Maladies Métaboliques, Laboratoire de Recherche PCIM, Université Qadi Ayyad, CHU Mohamed VI, Marrakech, Maroc

**Keywords:** Observance thérapeutique, HTA, diabète, antihypertenseurs, antidiabétiques oraux, insulinothérapie, treatment adherence, HT, diabetes, antihypertensive drugs, oral antidiabetic, insulinotherapy

## Abstract

Les diabétologues sont confrontés à des diabétiques le plus souvent hypertendus et polypathologiques, les rendant peu observants des différentes thérapeutiques prescrites. Le but de notre étude est d’évaluer l'observance au traitement chez les patients diabétiques hypertendus. Il s'agit d'une étude descriptive prospective transversale. La population est faite de patients diabétiques de type 2 hypertendus vus aux urgences, à la consultation ou hospitalisés au service d'Endocrinologie. L’échantillon est non probabiliste. La population est faite de 61 patients, d'un âge moyen de 48,9 ans avec une hypertension (HTA) de type systolique dans 59%, diastolique dans 16% et systolo-diastolique dans 25% des cas. Les patients sont équilibrés sur le plan glycémique dans 65% des cas, alors que l’équilibre tensionnel n'est atteint que dans 41% des cas. En ce qui concerne l'observance thérapeutique, 24% des patients sont observant totalement au traitement prescrit, 33% ne sont pas observant au traitement et 43% ont une observance partielle au traitement dont 73.68% préfèrent le traitement destiné au diabète et 26,32% prennent le traitement anti hypertenseur. Les facteurs altérant l'observance au traitement médicamenteux sont un surcoût du traitement un nombre élevé de médicaments prescrits, un nombre important de prises quotidiennes de médicaments, une intolérance au traitement et une mauvaise information du patient sur la justification du traitement. Enfin, les patients diabétiques hypertendus sont classés comme patients à haut risque cardiovasculaire ce qui impose l'application de stratégies thérapeutiques parfois lourdes. Cette polymédication entrave la qualité de l'observance thérapeutique des patients ayant une pathologie chronique dont la compliance au traitement constitue la pierre angulaire de la prise en charge, et ce pour plusieurs raisons en particulier d'ordre économique.

## Introduction

Parfois désignée sous les termes de compliance ou d'adhésion thérapeutique, l'observance correspond au respect des instructions et des prescriptions médicales. Elle reste un paramètre clé de la prise en charge des pathologies chroniques.

L'hypertension artérielle (HTA) est associée au diabète de type 2 (DT2) dans 80% des cas, elle contribue au risque élevé de maladies cardiovasculaires associée au diabète de type 2 [1]. En effet, les patients diabétiques sont souvent polyvasculaires, hypertendus et multitarrés ce qui oblige les médecins à prescrire des thérapeutiques complexes rendant difficile l'observance des multiples thérapeutiques prescrites.

Cette étude vise à analyser et évaluer l'observance thérapeutique par les patients diabétiques hypertendus polymédiqués. La particularité de cette étude revient à la nature de la population qui est faite de patients diabétiques hypertendus qui constitue une sous population de diabétiques alors que les études qui ont été déjà réalisées n'analyse qu'un seul volet de la pathologie qui est soit le diabète ou l'HTA. Il existe d'autres études qui évaluent l'observance thérapeutique chez les patients ayant d'autres pathologies chroniques d'ordre psychiatriques ou respiratoires par exemple. Ainsi le fait de ne pas comprendre l'importance du traitement prophylactique diminue l'adhérence du patient à son traitement et entrave, par conséquent, son observance.

## Méthodes

Il s'agit d'une étude prospective descriptive, réalisée de janvier 2011 à Mars 2011 prenant comme population des patients diabétiques de type 2 hypertendus vus en consultation ou hospitalisés au Service d'Endocrinologie, Diabétologie et Maladies Métaboliques du CHU Mohamed VI. Ont été exclus les patients ayant une HTA secondaire ou un autre type de diabète.

Tous les patients ont bénéficié d'une collecte de données via un questionnaire identifiant le patient, déterminant ses caractéristiques épidémiologiques et cliniques à savoir l'anciéneté du diabète, de l'HTA, les antécédents cardiovasculaires, les complications cardiovasculaires et précisant son suivi, son traitement, le nombre de comprimés par jour, et le traitement pris effectivement par le patient ainsi que les causes de non observances. Pour l’évaluation de l'observance, les six questions du “test de l′évaluation de l′observance” étaient celles d′un autoquestionnaire mis au point et validé par Girerd et al. (Table 1) [2]. Les patients sont jugés équilibrés pour DT2 et HTA sur la base des objectifs thérapeutiques personnalisés pour chaque patient selon les recommandations internationales. L'examen clinique précise le poids, la taille, l'indice de masse corporelle, le tour de taille, avec prise de la glycémie capillaire et la tension artérielle.

**Tableau 1:** Test d’évaluation de l'observance

Question	Reponses	
	Oui	Non
1. Ce matin avez-vous oublié de prendre votre médicament ?		
2. Depuis la dernière consultation, avez-vous été en panne de médicament ?		
3. Vous est-il arrivé de prendre votre traitement avec retard Par rapport à l'heure habituelle ?		
4. Vous est-il arrivé de ne pas prendre votre traitement parce que certains jours, votre mémoire vous fait défaut ?		
5. Vous est-il arrivé de ne pas prendre votre traitement parce que certains jours, vous avez l'impression que votre traitement vous fait plus de mal que de bien ?		
6. Pensez-vous que vous avez trop de comprimés à prendre ?		
Total des « Oui » :		

Réalisation du test : Au cours de la consultation le médecin demande au patient les questions ci-dessus.

Interprétation du test : Total des OUI = 0 Bonne observance; Total des OUI = 1 ou 2 Minime problème d'observance; Total des OUI ≥ 3 Mauvaise observance

## Résultats

La population a été faite de 61 patients dont 40 femmes soit 66% des patients contre 21 hommes soit 34% des patients. L’âge moyen des patients est de l'ordre de 52.38 ans avec un pourcentage de 18% de patients diabétiques âgés. 59% des patients avaient une HTA systolo-diastolique, 25% avaient une HTA systolique et 16% présentaient une HTA diastolique.

Concernant l’équilibre glycémique des patients, 65% des patients étaient bien équilibrés. L’équilibre tensionnel était assuré chez 41% des.

Pour l'observance thérapeutique, 24% des patients avaient une bonne observance au traitement, 33% étaient non observants et 43% étaient observants de façon partielle (c′est-à-dire n'adhèrent qu’à un seul type de thérapeutique, soit du diabète ou de l'HTA).

Concernant l'observance partielle, pour le traitement de l'HTA, 13% des patients y adhèrent. Quant au diabète, 87% des patients sont observants partiellement au traitement antidiabétique. Pour le type de thérapeutique de diabète chez ces patients, nous avons trouvé que 80% des patients mis sous traitement oral prennent régulièrement leurs traitements, par ailleurs, 44% des patients mis sous insuline sont observants.

En ce qui concerne le traitement préventif (antiagrégants plaquettaire et traitement hypolipémiant), il est prescrit chez 45 patients (soit 73,77% du total des patients) dont 30 patients (soit 67% des patients) sont non observant et 15 (soit 33% des patients) sont compliants à ce type de traitement.

Parmi les causes de la non observance thérapeutique dans notre contexte (Table 2) vient en premier le surcoût du traitement retrouvé chez 78,26% des patient, suivi par le nombre élevé de médicaments prescrits retrouvé chez 67,39% des patients puis le nombre élevé de prises quotidiennes chez 65,22% des patients et le statut socio-économique défavorable chez 65,39% des patients, l'absence de couverture médicale généralisée chez 54,35% des patients, la prescription de forme galénique non acceptée par le patient chez 32,61%, l'absence d'une bonne éducation ainsi que la mauvaise information du patient diabétique hypertendu sur la justification du traitement, la notion de petit diabète et d'HTA réversible retrouvé chez 21,74% des patients, enfin l'oubli de la prise médicamenteuse est décrit chez 2,17% des patients.

**Tableau 2:** Causes de non observance thérapeutique

Causes de non observance	Nombre de patient	Pourcentage
Surcoût	36	78.26%
Nombre élevé de médicaments prescrits	31	67.39%
Nombre élevé de prises quotidiennes	30	65.22%
Absence de couverture sociale généralisée	25	54.35%
Forme galénique	15	32.61%
Mauvaise information du patient sur la justification du traitement. Exemple: petit diabète	10	21.74%
Oubli	1	2.17%
Statut socio-économique défavorable	30	65.22%

## Discussion

Le patient diabétique hypertendu est un sujet à très haut risque cardiovasculaire et rénal. A ce titre, il doit bénéficier d'un traitement antihypertenseur précoce et intensif [3]. Outre les mesures hygiéno-diététiques, qui gardent une place cardinale dans la prise en charge, l'instauration sans délai d'un traitement antihypertenseur médicamenteux est recommandée [3] associé au traitement antidiabétique. Il faut donc instaurer des stratégies thérapeutiques souvent lourdes, impliquant l'association d'une grande variété de thérapeutiques, notamment le traitement antidiabétique oral ou insulinothérapie ou traitement mixte, les antiagrégants plaquettaires, bêtabloquants, dérivés nitrés ou agonistes potassiques, statines, inhibiteurs calciques, inhibiteurs de l'enzyme de conversion’. Tous ces facteurs peuvent entraver l'observance au traitement chez le patient diabétique hypertendu polymédiqué.

L'hypertension artérielle est le plus souvent associée au diabète de type 2 dans le cadre du syndrome métabolique, elle est moins fréquente dans le diabète de type 1 et survient généralement après le développement de l'insuffisance rénale [3]. Ces deux pathologies, associées dans 80% des cas, sont plus fréquentes particulièrement chez les personnes âgées, avec un pic situé entre 66 et 69 ans. Cette association HTA et diabète de type 2 est responsable d'une majoration du risque cardiovasculaire [4, 5]. Dans cette étude, 66% des patients sont de sexe féminin. La moyenne d’âge des patients est de l'ordre de 52.38 ans, les patients âgés constituent 18% de la population.

L'HTA la plus fréquente est essentiellement systolo-diastolique, l'HTA systolique isolée peut être également observée, avec des chiffres tensionnels plus élevés, ce qui est un facteur prédictif d’événements cardiovasculaires plus que dans l'HTA systolo-diastolique [6]. Cette étude montre que 59% des patients avaient une HTA systolodiastolique, 25% avaient une HTA systolique et 16% présentaient une HTA diastolique.

Seulement moins du tiers des patients hypertendus traités sont normalisés dans la plupart des pays [7]. La non-observance thérapeutique a été citée comme la raison principale de cet échec [8, 9]. En effet, environ 40% des hypertendus ne se conformeraient pas à leur traitement.

Les relations entre les chiffres tensionnels, en particulier l′hypertension normalisée et l′observance thérapeutique ne sont pas retrouvées dans une étude faite en côte d'Ivoire par Adoubi K. A et all [2]. Certains auteurs expliquent cette éventualité par le fait que les non répondants au traitement ne sont pas toujours des non observants [10] et aussi par l′effet “brosse à dents” [11]; les patients se brossent souvent les dents avant d′aller voir le dentiste. De même, les patients observent correctement leur traitement avant la visite médicale. En effet, la compliance s′améliore avant la visite médicale pouvant masquer une hypertension artérielle mal équilibrée entre les visites. Cette étude montre que l’équilibre glycémique est assuré chez 65% des patient, or l’équilibre tensionnel n’était assuré que chez 41% des patients à cause d'un arrêt des thérapeutiques prescrites soit d'une façon définitive ou avec reprise du traitement avant quelques jours de la date prévue de la consultation alors que les thérapeutiques antihypertensives ne sont, en général, pas efficace qu'après une prise régulière pendant deux semaines ou plus ce qui rend inefficace cette reprise thérapeutique évoquant ainsi des fausses résistance aux traitement antihypertenseur alors qu'il s'agit d'un simple problème d'adhérence au traitement.

On constate que 70% des traitements anti-hypertenseurs sont mal suivis. Chez les diabétiques, 50% des prescriptions médicales ne sont pas acceptées. Ce chiffre s’élève à 70% pour les règles hygiéno-diététiques [12]. Le patient qui a une observance partielle se distingue par le fait qu′il fait un effort pour participer à son traitement; il prend suffisamment de comprimés pour indiquer qu′il accepte le principe du traitement et qu′il y participe, mais il en omet ou en oublie suffisamment de telle façon qu′il perd tout le bénéfice du traitement [13]. L′oubli et le sentiment que le traitement n′est pas toujours nécessaire sont les deux causes d′observance partielle souvent retrouvées [11]. Avec près de 20% de mauvais observants et plus de la moitié des patients avec un minime problème d′observance, l′adhésion au traitement est peu satisfaisante selon une étude réalisée en Cote d'Ivoire [2]. Une étude européenne a relevé des résultats meilleurs, compte tenu des conditions sociales et économiques dont ils disposent à savoir la disponibilité d'une couverture sociale généralisée: dans une première étude 66% des patients avaient une bonne observance et 10% une mauvaise observance [14] or, dans une deuxième étude, 39% présentait une bonne observance et 8% une mauvaise observance [15].

Dans cette étude, 24% des patients avaient une bonne observance au traitement, 33% étaient non observant et 43% avaient une observance partielle. Parmi les patients observant partiellement au traitement, 13% sont observant au traitement de l'HTA et 87% sont observant au traitement du diabète!

Une autre étude réalisée en côte d'ivoire [15], elle avait montré que l'observance thérapeutique, chez une population d′hypertendus, est mauvaise dans leur contexte particulièrement chez ceux qui ont déjà des complications liées à l′HTA. L'indigence, et l'absence d'une couverture sociale généralisée, comme dans notre contexte, sont les principales causes de l’élévation du taux des patients non observant.

Les facteurs liés à la mauvaise observance sont multiples. Ils sont d′ordre médical, sociodémographique, économique mais aussi culturel et comportemental. Nous nous sommes intéressés, dans cette étude, à quelques uns d′entre eux facilement accessibles au cours d′une consultation. Le surcoût, le bas niveau socio-économique, la prescription de plusieurs médicaments et l′absence d′assurance, facteurs de mauvaise observance dans notre étude ont déjà été cités par d′autres auteurs [2, 11, 15].

La non-observance serait plus fréquente chez les patients plus âgés à cause de la diminution de leur autonomie et de l'installation plus ou moins importante de troubles cognitifs et humoraux en particulier la maladie d'alzheimer et la dépression; dans cette étude, la population âgé représente 18% des patients, c'est une population particulière qui se caractérise par la fréquence des complication chronique liées au diabète et à l'HTA, ces complication constituent ainsi une cause surajoutée de mal-observance au traitement [2]. Dans une étude menée dans notre formation en 2011 qui a analysé les caractéristiques de l'association du DT2 avec l'HTA chez le sujet âgé [1], Seulement 4,2% des patients avaient une HbA1c ‘ 6,5% avec une HbA1c moyenne à 9,7 ± 2,1%, 60% des patients avaient une HTA systolo-diastolique ainsi le contrôle de la pression artérielle n'a pas montré d'effet préventif chez cette population sur les complications micro et macro vasculaires (mémoire glycémique et rigidité artérielle) qui sont fréquentes chez cette population particulière caractérisée par une anciénneté relativement élevée du diabète et de l'HTA [1]. Au-delà de 75 ans, l'observance semble moins bonne [16, 17], si le traitement va à l'encontre des traditions religieuses ou culturelles du patient, le risque de non-observance est élevé [18]. Le statut socio-économique défavorable l'absence de travail, les difficultés de logement se corrèlent également d'une façon fréquente avec la non-observance. Les contraintes de prises médicamenteuses deviennent accessoires pour des patients en exclusion, soumis à des problèmes de précarité, d'alimentation, de toxicomanie’^17^. Au-delà des paramètres liés aux modalités de prise du traitement, la complexité du traitement inclut également l'ensemble des gestes associés: par exemple, la complexité du traitement par l'insuline du diabète a été soulignée, et l'observance peut être différente selon que l'on envisage l'injection de l'insuline, les mesures de la glycémie, ou l'adaptation de la dose d'insuline. C'est cette dernière qui fait défaut le plus souvent, anéantissant ainsi les efforts considérables (par exemple, quatre injections d'insuline et quatre mesures de la glycémie par jour) consentis par les patients dans la gestion d'un bon équilibre du diabète [18].

La galénique du produit est aussi à prendre en considération: gouttes mal dosées (tremblement ou erreur de comptage), sirops bus à la bouteille. N'oublions pas non plus le prix ou les conditions de remboursement qui peuvent constituer un obstacle pour certains patients [12].

La non-observance peut être aussi l'expression d'un mal-être [19] et amener à une prise en charge psychologique spécifique. Il existe aussi des mouvements de déni de la maladie [16]. La représentation que le patient se fait de sa maladie («j'ai un petit diabète») joue un rôle non négligeable dans le respect du traitement de même que ses croyances par rapport au traitement («on a prescrit de l'insuline à ma grand-mère et elle est morte»). L'information et la publicité conditionnent les réactions du patient au traitement et son comportement [20].

**Figure 1:**
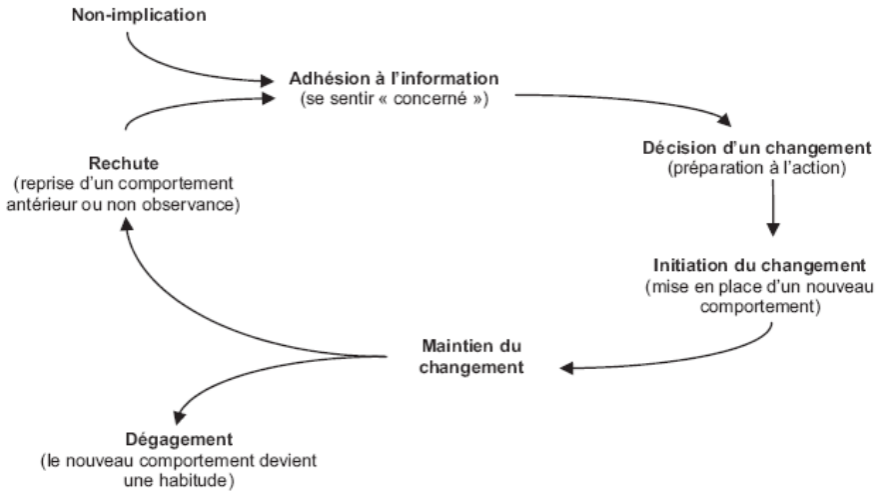
Le modèle de Prochaska et Di Clemente explique les étapes à suivre pour améliorer l'observance chez les patients, en passant d'un comportement de non observance à autre de bonne observance. Ce changement demande du temps et passe par plusieurs étapes qui commencent par l'explication facilitée de la pathologie et de ses complications éventuelles pour passer à l'explication de l'importance du traitement

L'amélioration de l'observance est un problème difficile et complexe. En effet, l'observance n'est pas un comportement toujours facile à adopter pour les patients [16] et, comme tout nouveau comportement, il demande du temps et passe par les étapes décrites dans le modèle de Prochaska [21] (Figure 1) et reprises dans le modèle PADIM (Figure 1) [21]; d'après cette théorie qui explique les étapes à suivre pour faire adhérer les patients aux traitements prescrits, on constate qu'il faut toujours passer par l'explication facilitée de l'information concernant la pathologie à traiter, les risques et les complications pouvant être liés à cette pathologie pour bien comprendre, par la suite, la nécessité et l'importance du traitement; à savoir le traitement symptomatique, étiologique et préventif pour décider de changer les habitudes habituelle du patient dès la première prescription médicale ou même en cas de non observance. Il faut également, dans le cadre des maladies chronique, tel le diabète et l'HTA, toujours expliquer la nécessité du suivi et du traitement à vie car ceci rendrait la prise médicamenteuse comme habitude irréversible et nécessaire dans la vie du patient.

**Figure 2:**
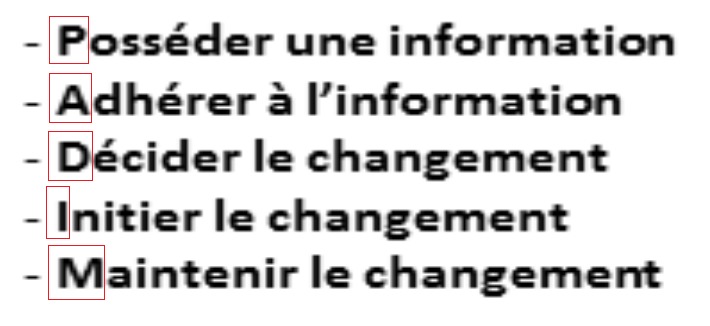
Le modèle de PADIM a repris les étapes décrites dans le modèle de Prochaska

L'amélioration de l'observance nécessite donc d'agir tout au long de la chaîne de soins, d'une part au moment de la consultation et d'autre part, parfois, dans des structures spécialisées d’éducation thérapeutique qui constitue, en diabétologie, une composante essentielle de la prise en charge, car, en effet, parmi les causes de la non compliance au traitement, on trouve la notion de l'incompréhension de la nécessité des thérapeutiques prescrites ou même de la pathologie traitée.

## Conclusion

La non-observance thérapeutique est un problème important de santé publique qui affecte autant l'espérance de vie du patient que le coût des soins de santé. Cette étude montre qu'une grande part des patients diabétiques hypertendus polymédiqués n'observent pas les thérapeutiques qui leur été prescrites surtout celles antihypertensives. Parmi les principales causes de non observance au traitement, dans notre contexte où dominent l'analphabétisme et la pauvreté, vient en premier le surcoût du traitement, le nombre élevé des médicaments, l'absence de couverture sociale généralisée, et le manque d'information du patient! Pour pouvoir remédier à ce problème de santé publique, il faudra donc agir sur les éléments relativement faciles à gérer n'ayant pas d'impacte économique sur le patient. Une sensibilisation des médecins sur l'importance du temps à accorder à l’éducation simplifiée et globale des patients, ainsi que la prescription de médicaments combinés de préférence de type génériques, en générale moins chers, ainsi il faudra inciter les patients d'adhérer au nouveau régime d'assistance médicale (RAMED) nouvellement instauré dans notre royaume visant l'amélioration et l'extension de la couverture médicale qui constituera un des pilier du développement humain et social. Le problème de la non-observance thérapeutique n'est pas neuf; Hippocrate affirmait déjà que «les patients mentent souvent quand ils disent prendre leurs médicaments». La solution miracle n'existe pas, mais une réflexion abordant les causes de ce phénomène vieux comme le monde devrait permettre de progresser dans sa résolution.
